# Improving the performance of the coffee supply chain using integrating fuzzy MCDM and simulation methods

**DOI:** 10.1371/journal.pone.0326581

**Published:** 2025-06-18

**Authors:** Ghazi M. Magableh, Abdullah F. Al-Dwairi, Ahmad Alhamouri

**Affiliations:** 1 Industrial Engineering Department, Yarmouk University, Irbid, Jordan; 2 Industrial Engineering Department, Jordan University of Science and Technology, Irbid, Jordan; Istanbul University: Istanbul Universitesi, TÜRKIYE

## Abstract

Coffee is one of the drinks that are consumed worldwide. The global coffee beans industry is currently facing several challenges, including The COVID19 pandemic’s long-term effects on their supply chains (SCs), adverse weather conditions affecting major coffee-producing regions, escalating price dynamics, and the increased in transportation costs. Jordan significantly relies on coffee imports, a critical agricultural product that constitutes an important part of local trade. Due to the lack of previous studies, the research aims to evaluate the current SC and improve Jordan’s coffee SC performance by reducing lead time and shipping cost. The research first uses the combined Fuzzy Analytic Hierarchy Process (FAHP) and The Fuzzy Technique for Order of Preference by Similarity to Ideal Solution (TOPSIS) method to rank the supplier and choose the best suppliers based on established criteria. Then, the research proposes an analysis with the goal of enhancing the efficiency of Jordan’s coffee SC by applying Discrete Event Simulation (DES). Real company data is utilized to study, apply and evaluate the methodology used. The decision-making process employ decision makers and expert opinion input, providing a comprehensive and balanced evaluation of suppliers. Different scenarios are evaluated and compared based on the integrations of the FMCDM results in the simulation model. The findings identify the best and least favorable suppliers, highlighting Ethiopia’s leadership in several indicators. The outcomes suggest reducing lead time and shipping costs, investing in technology, and establishing a culture of continuous improvement to improve efficiency, stability, and adapt to market conditions. The research provides a comprehensive analysis of the current state of the coffee SC and suggests opportunities for development. Finally, recommendations to improve the current coffee SC in Jordan and directions for future research are discussed.

## Introduction

The current business and industry landscape is undergoing a fast transition. The ability to swiftly change Supply Chains (SCs) for product acquisition, manufacturing, and distribution is no longer a one-time event [1,2]. With fierce competition, rising costs, immediate consumer demands, and unpredictable shifts in demand patterns [2,3], it is critical to imagine a SC that can adapt its products, processes, and systems to changing conditions. To address these expanding issues efficiently, SCs must adopt intelligence and technology [2]. Current public health issues have focused on the complicated nature of the food SC. It is becoming increasingly clear that in the coming years, there will be greater oversight and improved governance in the establishment and operational planning of food SCs, particularly for consumables intended for human feeding, such as agricultural products. This trajectory implies that conventional SC techniques will be reassessed and possibly recalibrated in near future [4].

Jordan significantly relies on coffee imports, a critical agricultural product that accounts for a significant amount of its trade. The year 2021 had a considerable import value of roughly $110 million for coffee products, highlighting its economic importance [5]. The global coffee industry is currently facing several challenges, including the COVID-19 pandemic’s long-term consequences on SCs. SCs, adverse weather conditions affecting major coffee-producing regions, escalating price dynamics, and increased transportation costs [6]. In order to improve the overall performance and sustainability of the coffee supply chain in Jordan, this research uses a distinctive combination of fuzzy Multi-Criteria-Decision-Making (MCDM) and DES to improve the coffee supply chain.

DES is a computational modeling method used in systems engineering and operations research to study the dynamic behavior of complex systems with discrete, isolated events. It helps record and examines the movement of entities across different chain phases while considering discrete events affecting system behavior [7]. MCDM is a methodical and analytical technique used in decision making and management to select the best option from a range of competing criteria or objectives [8].

An important attribute of supply chain management, supplier selection, has a big influence on overall performance. Supplier selection can be developed by the FAHP-FTOPSIS approach, which enables decision-makers to efficiently control the ambiguity and uncertainty of their conclusions. FAHP is an advanced MCDM technique that addresses the subjectivities and uncertainties present in human judgment by combining fuzzy logic with the conventional AHP. FAHP has been used by numerous researchers to select the best supplier in the SC. For instance, Ramos et al, (2020) used a multicriteria decision model depend on the FAHP technique to choose suppliers for a Brazilian food company [9]. Also, Wang et al, (2019) propose the use of an MCDM model to evaluate and choose suppliers. Experts outline every criterion that influences the evaluation and selection of suppliers. The weight of each criterion was then found out using FAHP comparative analysis method [10]. Additionally, Ersoy et al, (2021) used the AHP method to select the best supplier for a food production company from three competitors, with four purchasing department specialists providing their evaluations [11]. TOPSIS is an MCDM system that focuses on selecting alternatives with both the longest and the shortest geometric distances between the negative ideal solution and the positive ideal solution. It involves forming a decision matrix, normalizing it, and multiplying the values by the criteria weights, ensuring effective and efficient selection of prominent class criteria. TOPSIS is utilized in various fields like procurement decisions, supplier selection, and material selection.

Table 1 summarizes the related previous studies concerning the coffee supply chain. It illustrates the differences between the current study and previous studies. The table illustrates the focus of each study, tools and methodologies used, and the citation of the references. The current research is distinct from others as it considers the combined fuzzy AHP and fuzzy TOPSIS methods, the integration of the FMCDM within the DES model to study, evaluate, and improve the JCoSC.

**Table 1:** Summary of the CoSC studies.

Focus	Tools/ Methods	Reference
Coffee supply chain performance measurement	Supply Chain Operations Reference model (SCOR)	[12]
Solution for the supply chain in the coffee industry	Blockchain-based traceability	[13]
Classify and identify the cooperatives’ participants in the coffee supply chain	Analytical	[14]
Identify the coffee supply chain network from farmers to consumers	Analytical &statistical	[15]
Analyze the supply chain system of Robusta coffee	Descriptive	[16]
Modeling the distribution of organic coffee supply chain	Empirical and conceptual framework	[17]
Coffee supply chain analysis	Descriptive statistics	[18]
Enhancing coffee SC towards sustainable growth with big data and modern agricultural technologies	Analytical	[19]
Analysis of critical knowledge in a coffee SC	Analytical &statistical	[20]
Forecasting model for the coffee bean supply chain	Nonlinear grey Bernoulli model	[21]
Modeling of Gayo Arabica coffee industry SCM	Simulation	[22]
Sustainable development for a coffee supply chain	Simulation	[23]
Blockchain modeling for traceability in supply chain of coffee agroindustry	Analytical and modeling	[24]
Decision support system for reactive aggregate production scheduling in the green coffee SC	Agent-based simulation model driven decision support system	[25]
Resilient supply network design: disruptive events modelling for the coffee industry in Colombia	Mixed integer programming model and discrete event simulation	[26]
Assessing the income effects for coffee farmers	Agent-based simulation	[27]
Enhancing the competitive advantages of coffee SC	Causal loop method	[28]
Supplier selection in the coffee bean supply chain	FAHP, VIKOR	[29]
Selecting instant coffee supplier for supermarket	AHP	[30]
Ranking suppliers and selecting the best Coffee supplier using combined FMCDMs techniques and enhancing the efficiency of coffee supply chain	Integrated FAHP-FTOPSIS & Integrated FMCDM within the discrete event simulation model	This research

The combination of AHP and TOPSIS methods is commonly used in multi-criteria decision-making due to their complementary nature. AHP determines the importance of criteria, while TOPSIS ranks alternatives based on those weighted criteria. Combining these methods improves results due to the eigenvector concept in AHP and the weighting criteria processed by TOPSIS. Both methods and their integration have been used in supplier selection in many previous studies to benefit from the advantages of their combination to increase decision-making efficiency. Therefore, the FAHP-FTOPSIS approach is considered appropriate for the coffee supplier selection.

By combining FMCDM and DES methodologies, the Jordanian coffee SC can be explored, providing industry with crucial data and insights in a rapidly changing market. Based on the available information and to our knowledge, there is no study that has investigated the coffee supply chain in Jordan. Therefore, the research seeks to answer the subsequent questions: What are the constraints to the coffee supply chain in Jordan? Who is the best coffee suppliers to Jordan based on the current position? How can shipping costs and the time required to ship coffee from the source be reduced? How to optimize the type, size, and number of containers? And how can the performance of the coffee SC be improved? The research’s main goal is to study and suggest a cutting-edge strategy for improving the efficiency of Jordan’s coffee supply chain. The research’s specific objectives are to:

1. Investigate and evaluate the current state of the coffee SC in Jordan including the analysis of the current suppliers, SC processes and operations, and SC network.
2. Select the best coffee suppliers to the Jordanian market and rank the suppliers based on fuzzy MCDM technique.
3. To test and evaluate the different proposed SC configurations, flows, and processes using simulation modeling to improve the SC performance.

The study focuses on the Jordanian coffee beans supply chain. By using a comprehensive approach, it seeks to fully comprehend the factors and challenges that affect the supply chain’s overall performance by capturing the subtleties and interconnections within each SC stage. The main contribution is to provide a customized, data-driven strategy for improving Jordan’s coffee supply chain (JCoSC). The study ranks and evaluates coffee suppliers using the fuzzy-AHP -TOPSIS model, making it possible to choose the best coffee source for the Jordanian market. The combination of FMCDM techniques within DES model allows for a comprehensive framework for decision-making that considers the present situation of the supply chain, the range of factors at play, and the unique circumstances of the Jordanian market. The outcomes are expected to make a substantial contribution to the academic field of supply chain management as well as offer practical advice to industry professionals, decision-makers, and other parties with an interest in the Jordanian coffee market. The study is significant for the research community as well as the JCoSC specialists, as it will offer a useful tool to assess the SC’s current state and make improvements quickly by evaluating various scenarios and offering practical recommendations.

The paper is structured as follows: section 2 analyzes the previous studies related to the food SC, Coffee SC, SC in Jordan, SC simulation and Coffee SC, MCDM used in the SC and Coffee SC, and gaps and contributions. Section 3 discusses the methodology used. Section 4 Rank and select the best coffee supplier using FAHP-FTOPSIS, section 5 introduces the simulation model and the different scenarios used to compare and improve the current coffee SC. Section 6 explain the results and Section 7 proposes the conclusion and recommendations.

## Literature review

Coffee is considered one of the main consumer goods in Jordan, especially Arabic coffee, which is consumed throughout the day and on special and public occasions. Arabica, Robusta, Liberica, and Excelsa are the four main coffee types used in Jordan, each with different taste profiles. Coffee has been part of authentic Jordanian customs since early times, as is the case in the Middle East and North Africa area in general. It carries tribal and social connotations related to generosity, chivalry and gallantry. Therefore, coffee is considered one of the basic goods in Jordan, which has its connotations and symbolism as a drink of social value. Coffee kiosks and shops have spread remarkably in Jordan. These kiosks have become the focus of attention for travelers on the external roads between the main Jordanian cities and in the residential neighborhoods of the residential areas and tourist places. Statistics indicate that there are thousands of shops serving coffee drinks within cities, villages, and the main roads between cities [31]. The coffee business in Jordan does not require a large capital. Anyone who has a thousand dollars at most can build a kiosk out of tin or iron and bring the coffee selling supplies such as coffee pots, a heater, and other supplies for making coffee, and then start selling [31]. Consequently, the coffee trade has been active since ancient times, and interest in the supply chain and sources of purchasing coffee has increased, especially some desirable types. The sustainability and profitability of the global coffee industry are contingent upon the effective management of supply chains. Therefore, it has become necessary to focus on the coffee supply chains (CoSC) as they have a significant influence on SC decision-making, process optimization, and sourcing the best suppliers.

Worldwide, a large number of studies have looked into the food supply chain. For instance Vostriakova et al, (2021) suggested the enhancement of the agri-food logistics distribution system and offered scientific validation of theoretical and methodological principles. The framework includes gap and process analysis, value stream mapping (VSM), validation and improvement areas definition, and imitation modeling [32]. Additionally, Nakandala et al, (2016) created a technique to help logistics managers improve the fresh food supply chain and make cost-effective transportation decisions. The goal is to minimize overall costs while preserving food product quality above acceptable limits [33]. As part of the agri-food SC, a comprehensive research has been conducted to analyze the coffee SC, these studies contribute significantly to product quality improvement and SC performance. Nguyen et al., (2021) conducted a study that focused on both internal and external variables that impede the efficacy of Vietnam’s coffee SC. The Supply Chain Operations Reference (SCOR) model was used to measure SC performance in the coffee industry [12]. Building on the insights gained from Nguyen et al.’s comprehensive analysis, Alamsyah et al, (2023) created a blockchain-based traceability solution for the supply chain of the coffee industry. A flexible model that can be modularly adapted to various supply chain scenarios in the coffee industry is proposed. It shows how to modify the overall model’s stakeholder count to fit specific industry needs in order to apply the model to that industry [13].

As a country that depends on imports for many agricultural, food and industrial products, supply chains in Jordan play crucial role in the quality and volume of trade, the availability of goods and their prices. Gaining a better understanding of Jordan’s supply chain dynamics have a great impact on the country’s economy by increasing competitiveness in the market, reducing operating costs, and enhancing trade efficiency. Several studies and research on various aspects of supply chains in Jordan highlights the challenges and suggest solutions for enhancing supply chain performance. Magableh et al. (2024) developed comprehensive and impactful strategies to address supply chain instability, particularly considering the intense competition and rapid advancements in business, economics, and technology. The strategies are ranked by priority and implementation order using the fuzzy PROMETHEE approach [34]. Magableh (2021) studied the interruptions, accompanying difficulties, and trend of the COVID19 pandemic’s effects on SCs. An examination of the stages, phases, and manifestations of SCs was carried out concerning the outcomes, prospects, and advancements brought about by the pandemic [35].

MCDM has been used in decision making in different fields and in various disciplines, for example: Using fuzzy VIKOR for rankings of India’s states determined by sustainable women’s empowerment [36], WASPAS approach for choosing a location for a university campus girls’ hostel [37], CRITIC and COPRAS for ascertaining the best site for the university campus canteen [38], and The AHP-TOPSIS combination for choosing the best supervisor based on the student’s desired requirements [39].

The utilization of MCDM in supplier selection has been a focus of supply chain management research assisted manufacturers in selecting their best suppliers, demonstrating the utility of MCDM in specific industrial contexts such as electronics manufacturing [40], presented fuzzy MCDM techniques, suggested a ranking model for Jordan rice suppliers, and underlined the need of choosing and managing appropriate suppliers to satisfy customer demands. Within a predetermined framework, an integrated model was proposed for the selection of rice suppliers, considering variables about the pre-, during-, and post-selection processes [41]. Other research created a recovery strategy framework that explains the relationships at various stages between the primary problems caused by COVID19 in the SC, recovery choices, development areas, the goals of the recovery strategies and the strategic recovery plan to rebuild the SC. Combinations of quantitative and qualitative approaches are used to rate possible choices and identify possible opportunities for supply chain improvement. To rank and prioritize the areas that require improvement, a fuzzy TOPSIS technique combined with a fuzzy ANP technique is recommended. The results imply that supply chains can only be resilient to interruptions and different changes in the present and future disruptions by digitalization and utilization of emerging technology [42].

The use of MCDM tools in supplier selection is a widespread practice among researchers seeking to improve decision-making processes. MCDM methods was used to evaluate and rank supplier for example, FAHP to evaluate the supplier’s involvement in food safety and halal criteria [43], AHP and TOPSIS to rank suppliers of coffee beans based on weighted criteria [44], an integrated Weighted Aggregated Sum Product Assessment (WASPAS) and the Fuzzy-ANP model to choose the correct supplier [45], an integrating Multi-criteria Optimization and Compromise Solution method and FAHP to evaluate suppliers in the Coffee Bean Supply Chain [29], and AHP approach to evaluate and choose the best source for coffee [30]. Previous studies enhance supplier selection procedures by emphasizing the importance of adapted approaches, improved decision-making tools, and industrial context considerations.

The incorporation of simulation approaches in SC development plays a pivotal role in enhancing overall operational efficiency. Such approaches enable firms to gain a holistic view of their operations, streamline procedures, reduce potential risks, and create strategic decisions that result in overall SC efficacy enhancement. Numerous research projects have dedicated efforts to leveraging simulation models to improve SC dynamics. Singh et al, (2021) developing a public distribution system network simulation model. The model, featuring three distinct scenarios, illustrated SC disruption in the food industry. Notably, the integration of warehouses emerged as beneficial, enabling the fulfillment of demand from a backup warehouse if there are disruptions at the designated warehouse [46]. In a complementary vein, Piqueiro et al. (2022) developed a simulation framework that combines research on disruptive scenarios and resource allocation models to enhance decision making process. Their research focused on supply fluctuation and demand unpredictability, particularly in fire settings and considers supply variability and demand uncertainty brought on by disruptions [47].

As far as we know, no prior study has addressed the coffee supply chain in Jordan, including the analysis of the current suppliers, SC procedures, and SC network. The research is motivated by the lack of fuzzy MCDM models for evaluating coffee suppliers and choosing the best source for the Jordanian market and the lack of comprehensive simulation models to test and assess various coffee SC configurations and flows in an attempt to improve the coffee’s SC performance. Although the coffee supply chain, discrete event simulation, and multi-criteria-decision-making have all been the subject of several research efforts, none have combined these approaches with the goal of maximizing the performance of the coffee supply chain. This disparity emphasizes the need for a model that integrates these cutting-edge methods to deliver useful insights and advancements in the coffee SC.

To close this gap, this research pursues three main goals: first, it analyzes and assesses the current condition of the Jordanian coffee supply chain, including a detailed evaluation of suppliers, processes, and the supply chain network; second, an MCDM method that uses an fuzzy AHP-fuzzy TOPSIS technique to rank and select the best coffee suppliers for the Jordanian market is utilized; third, simulation modeling is employed to test and assess various supply chain configurations and process flows in order to improve performance. The integration of Fuzzy MCDM results and simulation modeling will provide valuable solutions for enhancing Jordan’s coffee supply chain.

## Methodology

**Fig 1:**
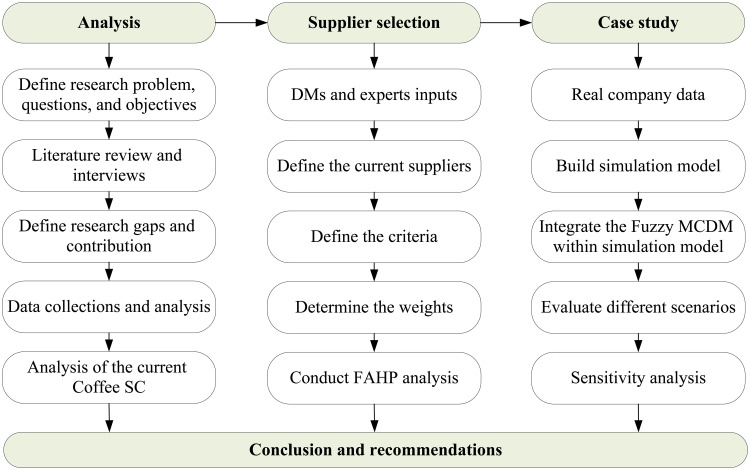
Research Methodology.

Fig 1 shows the study methodology which comprises four key parts including the analysis of the current supply chain and data collection, the application of fuzzy MCDM to rank and select the best coffee suppliers for Jordan, a simulation model with different scenarios to increase the performance of the SC, and the results and recommendations.

Real data is used from one of the major companies that import coffee to Jordan. This company is well-known in Jordanian popular circles. Then, the main suppliers of coffee to Jordan are identified and the countries from which coffee is imported were determined. A questionnaire is used and distributed to a number of DMs and experts in the field of the CoSC in Jordan. The study got approval from the Institutional Review Board (IRB) at Yarmouk University with reference nomber IRB/2024/476. Each participant received an informed consent form to take part in the study, and prior signed consent was acquired. Over the course of October 25 to December 10, 2024, the questionnaire was delivered, and responses and permissions were gathered. The team of experts consisted of ten senior employees in the coffee company, academics, experts in the supply chain, and official from government agencies concerned with import regulations, specifications, and customs. The data obtained from the survey results were used as inputs for FAHP-FTOPSIS to rank and determine the best suppliers.

In the second phase (suppliers ranking and selection), the integrated FAHP-TOPSIS will be used to rank suppliers and select the best Coffee supplier based the decision variables. Fuzzy TOPSIS is a decision-making method that combines TOPSIS with fuzzy logic to handle uncertainty and imprecise data in multi-criteria decision-making. It allows trade-offs between criteria, providing a more realistic form of modeling than non-compensatory methods. Fuzzy TOPSIS is a popular method for finding ideal solutions among similar options and automating the process to overcome ambiguity and uncertainty in the selection process. FAHP is a decision-making method that addresses uncertainty and ambiguity in traditional AHP. It allows for subjective judgments and a flexible scale, making it more realistic. FAHP is a combination of fuzzy mathematics and AHP and is particularly useful for multi-attribute analysis and structured hierarchy decision situations, providing priority weight vectors better than traditional AHP.

Next, the simulation model will be used to evaluate the current status of the coffee SC by using real data from the company. The data related to the quantities and times of imports, the quantities of imports from each supplier, and information about the supply chain including shipping, storage and distribution in addition to costs and lead times. After validation and verification of the simulation model, the results are analyzed and the current status of the supply chain will be explored.

In the third phase, Jordan’s coffee supply chain is investigated by combining the MCDM within DES model. The results from the previous phase, specifically the ranking of suppliers according to their importance, will be applied to a simulation model to analyze the coffee supply chain and demonstrate its impact on the performance metrics used. The characteristics and effect on the supply chain will then be identified and evaluated. Furthermore, a number of scenarios will be implemented and tested to evaluate if they improve the efficiency and performance of the JCoSC.

As the global coffee market grows, supply chain processes must be improved to remain competitive and meet evolving customer demands. The methodical approach is used to investigate, assess, and enhance the coffee supply chain in the unique context of Jordan. It also offers a road map for implementing DES and FMCDM approaches in the SC process. The integration of these methods provides a comprehensive and insightful analysis of supply chain dynamics, offering a unique perspective on decision-making [48].

The study presents a unique data-driven strategy for enhancing Jordan’s coffee supply chain, utilizing fuzzy AHP-fuzzy TOPSIS and DES models to rank and evaluate coffee suppliers, providing practical advice for industry professionals in the Jordanian coffee market.

## The integrated FAHP-FTOPSIS approach

MCDM involves multiple evaluation criteria of decision-makers’ opinions, where the ideal answer is determined by considering multiple factors [49]. Fuzzy theory is utilized in decision-making situations due to the ambiguity and uncertainty caused by varying experiences, attitudes, and knowledge of decision-makers. This approach optimizes MCDM procedures by addressing the vagueness and doubt in the decision-making process [50].

**Fig 2:**
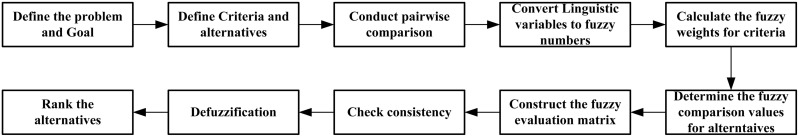
Steps of the combined FAHP-FTOPSIS calculations.

Because of its ease of use and capacity to handle the majority of decision maker issues, the AHP technique is one the most commonly utilized MCDM approach. The pairwise comparison is the main component of the AHP procedure. Weighing the options in light of several evaluation criteria pertaining to the professional opinions leads to the decision. The FAHP is introduced because the normal AHP cannot handle the subjectivity and inaccuracy of the pairwise comparison. When using a fuzzy approach, a trapezoidal or triangular fuzzy number is utilized in place of a crisp value to account for the decision-makers’ uncertainty and imprecision. Fuzzy numbers explain how the alternatives under examination behaved in relation to the chosen criteria [51]. Compared to the triangular membership function, the fuzzy trapezoidal (TPZ) membership function is more descriptive and more accurately covers the fuzzy universe. The methodological steps and the associated equations of the Fuzzy AHP-Fuzzy TOPSIS, adopted from [52], were applied in this research. Fig 2 shows the steps of FAHP-FTOPSIS calculations.

The following steps summarize the integrated methodology adopted from [52]

1. Determine the decision elements including the goal, alternatives, and decision-variables.
2. Establishing the comparison matrix using FAHP method:

$$\tilde{R}=\;[\begin{array}{ccc} {\tilde{R}}_{11} & {\tilde{R}}_{12}\ldots & {\tilde{R}}_{1n}\\ {\tilde{R}}_{21} & {\tilde{R}}_{22} & {\tilde{R}}_{2n}\\ {\tilde{R}}_{n1} & {\tilde{R}}_{n2} & {\tilde{R}}_{nn} \end{array}]$$ (1)

Where, ${\tilde{R}}_{ij}$ is the measure of $U_{i}$ compared with $U_{j}$, and $({{\tilde{R}}_{ij})}^{-1}$ is the measure of $U_{j}$ compared with $U_{i}$ (inversion). $U_{i}$ denotes the rate in the *i*^th^ row, and $U_{j}$ denotes the rate in the *j*^th^ column.


$${\tilde{R}}_{ij}=(m_{ij},n_{ij},o,p_{ij})$$ (2)

$$\left({{\tilde{R}}_{ij})}^{-1}=({p_{ij}}^{-1},{o_{ij}}^{-1},{n_{ij}}^{-1},{m_{ij}}^{-1}\right)$$ (3)

1. Applying the following formulas to calculate the consistency ratio (CR)

$$\begin{array}{c} CI=\;\frac{\left(\lambda_{\max}-n\right)}{\left(n-1\right)} \end{array}$$ (4)

$$CR=\;\frac{CI}{RI}$$ (5)

Where,  CI represent the consistency index, n is the matrix size, RI is the random index, and $\lambda_{\max}$ is the greatest eigenvalue of the matrix. Where the matrix is regarded consistent if the CR is smaller than 0.1.

1. Next, formulas 6 and 7 are used to compute the weights of the criteria by calculating 𝛼, 𝑗, 𝛽, 𝛼, 𝛾, 𝛽, 𝛾, and 𝛿.

$$\alpha_{j}=\;{\left[\prod_{j=1}^{n}m_{ij}\right]}^{\frac{1}{n}},\beta_{j}=\;{\left[\prod_{j=1}^{n}n_{ij}\right]}^{\frac{1}{n}},\gamma_{j}=\;{\left[\prod_{j=1}^{n}o_{ij}\right]}^{\frac{1}{n}},\;\delta_{j}={\left[\prod_{j=1}^{n}p_{ij}\right]}^{\frac{1}{n}}\;$$ (6)

$$\alpha =\;\sum_{j=1}^{n}\alpha_{j},\;\;\beta =\;\sum_{j=1}^{n}\beta_{j},\;\;\gamma =\;\sum_{j=1}^{n}\gamma_{j},\;\;\delta =\;\sum_{j=1}^{n}\delta_{j}$$ (7)

Then, get the geometric mean value ${\tilde{r}}_{j}=(\alpha_{j},\;\beta_{j},\gamma_{j},\delta_{j})$, and compute the inverse of the 𝑟𝑗̃ using Eq. 8.


$${{\tilde{r}}_{j}}^{-1}=(\delta^{-1},\gamma^{-1},\beta^{-1},\alpha^{-1})$$ (8)

After that, equation 9 is used to determine the weight of each criterion.


$${\tilde{y}}_{j}=\left(\alpha_{j}\delta^{-1},{\beta_{j}\gamma }^{-1},{\gamma_{j}\beta }^{-1},\delta_{j}\alpha^{-1}\right);j\in \left\{1,2,\ldots .,n\right\}$$ (9)

Lastly, the one row matrix representation of the fuzzy weight vector ˜Y is as follows:


$$\tilde{Y}=\;\left[\tilde{Y1}\;\;\;\tilde{Y2}\;\;\ldots \;\;\;\;\;\tilde{Yn}\right]$$ (10)

1. The establishment of the fuzzy evaluation matrix for the suppliers using FTOPSIS. The decision group’s evaluation of the supplier performance value ${\tilde{s}}_{j}$ of the element ${\tilde{s}}_{j}$ can be determined by:

$${\tilde{s}}_{j}=(\;\frac{1}{k})({\tilde{s}}_{j}1+{\tilde{s}}_{j}2+\ldots +{\tilde{s}}_{j}k)$$ (11)

Where, ${\tilde{s}}_{j}$ = (m, n, o, p) is a Fuzzy number indicating the evaluating significance of the attribute $s_{j}$, set by expert *k*, and 1 ≤ j ≤ n. Also, the Fuzzy evaluating-matrix can be obtained as;


$$\tilde{s}=\left[{\tilde{s}}_{1}\;\;\;\;{\tilde{s}}_{2}\;\;\;\ldots \;\;\;{\tilde{s}}_{n}\right]$$ (12)

1. The fuzzy evaluating matrix and the criterion weights are then used to determine the fuzzy evaluating vector for each supplier using eq. (13).

$$\tilde{Z}=\left[\frac{\left({\tilde{y}}_{1}\times {\tilde{s}}_{1}\right)+\left({\tilde{y}}_{2}\times {\tilde{s}}_{2}\right)+\;\left({\tilde{y}}_{3}\times {\tilde{s}}_{3}\right)+\ldots +\left({\tilde{y}}_{j}\times {\tilde{s}}_{j}\right)}{\left({\tilde{y}}_{1}+{\tilde{y}}_{2}+{\tilde{y}}_{3}+\ldots +{\tilde{y}}_{j}\right)}\right]$$ (13)

1. Defuzzification. Eq.14 is used to convert trapezoidal fuzzy numbers (TpFNs) into a crisp value.

$$Z=\;\frac{\left(m+2n+2o+p\right)}{6}$$ (14)

The decision makers (DMs) and experts team evaluates the coffee suppliers based on the selected criteria. The weight (relative importance) of each criterion is determined using a linguistic expression. Then the linguistic values (LVs) are converted to a 1–9 scale [52]. Table 2 summarizes these linguistic statements together with their corresponding crisp and trapezoidal scale.

**Table 2:** The TpFNs and their linguistic expressions for the criteria weights.

Relative importance (crisp value)	TpFN	Linguistic variable
1	(1, 1, 1, 1)	Equally significant
3	(2, 5/2, 7/2, 4)	Moderately significant
5	(4, 9/2, 11/2, 6)	Essentially significant
7	(6, 13/2, 15/2, 8)	Highly significant
9	(8, 17/2, 19/2, 9)	Extremely significant
X = 2, 4, 6, 8	(x-1, x – (1/2), x + (1/2), x + 1)	Intermediate values
1/3, 1/5, 1/7, 1/9	N/A	Inverse comparison values

The experts evaluate alternatives with respect to the criteria used in line with the Fuzzy TOPSIS technique using their knowledge and experience. The evaluation matrix considers a 5-point rating system, varying from very poor to very good. The TpFN associated with these linguistic values (LVs) are shown in Table 3.

**Table 3:** The LVs and their related TPZ Fuzzy numbers used to evaluate alternatives.

Linguistic variable	The related TpFNs
Very Good (VG)	(7, 8, 9, 10)
Good (G)	(5, 6, 7, 8)
Moderate (M)	(3, 4, 5, 6)
Poor (P)	(1, 2, 3, 4)
Very Poor (VP)	(0, 1, 2, 3)

## JCoSC simulation system

DES effectively addresses coffee supply network complexity by simulating stochastic behaviors, leading to thorough analysis, detection of bottlenecks, and assessment of interventions to maximize effectiveness in CoSC applications [53]. Arena simulation software offers a user-friendly platform for simulating CoSC complexities, offering a vast library of pre-built modules and large dataset management capabilities. Its detailed data analysis enhances communication and understanding of system dynamics [54]. This research utilizes DES and Arena simulation software to simulate JCoSC, ensuring efficiency and cost-cutting. DES allows for accurate outcome prediction and optimization, while Arena’s sophisticated features enable testing of techniques. This data-driven approach enhances supply chain management and decision-making, reducing lead times and enhancing overall efficiency.

Jordan’s coffee supply chain, like many others, is susceptible to a range of variations and unpredictability that could jeopardize its effectiveness. With DES, one may replicate the entire supply chain process—from procurement to distribution—under various conditions. Arena’s advanced features will make it possible to forecast outcomes more accurately, optimize processes, and evaluate different scenarios. This strategy is essential for reducing costs and lead times while raising the overall effectiveness of the CoSC. The application of DES using Arena simulation software in this study not only provides comprehensive analysis but also provides a strong foundation for data-driven decision-making that will improve supply chain management.

The supply chain for coffee is modeled with the main components including suppliers, distributors, carriers, and delivery locations. Taking into consideration that the local agent imports coffee from several sources at the same time, this is due to price changes, crop shortages, and other difficulties that affect imports. Real-world complications are incorporated into the definition of logical constructs, such as queues, processes, and resources, and disruptions to replicate the movement of coffee goods through the supply chain. Charts, animations, and output reports are examples of visual aids that helped make it easier to understand how different interventions affected the supply chain for coffee.

### Simulation model inputs

The data used in the study are based on information available through literature reviews, real case study data of the company under investigation, data from official and governmental bodies related to coffee bean imports and its supply chain, and reliable global sources in this field. The study, also, utilized official platforms Hapag Llyod and Trademap to gather data on expected shipping time and costs between coffee sources and Jordan, aiming to understand the dynamics of the coffee supply chain [55]. Official sources specializing in commerce and logistics provided the estimated lead time. Furthermore, credible sources offered details on shipping charges, describing the expenses related to delivering coffee from the supplier to the Jordanian market [5]. For up-to-date and precise information on Jordan’s coffee imports, official government websites providing trade statistics and import records were examined. These sources offer a thorough analysis of the quantity, and countries of origin of coffee imported into Jordan, and important insights into the market [5, 55].

**Fig 3:**
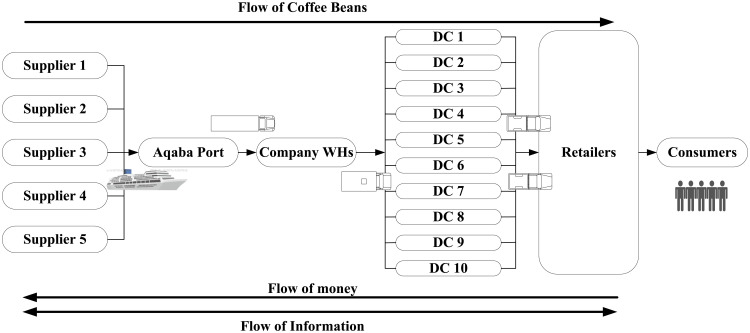
Coffee supply chain flow chart.

A well-known and major Jordanian coffee company was selected for the purpose of using its data in the study and analysis processes in this research. The company import, ships, stores, prepares and distributes coffee to retail and wholesale sellers throughout the Kingdom. The name of the company will remain confidential to maintain the secrecy of information and the privacy of the company. Therefore, the term “the company” will be used instead of the actual company name. To create a successful simulation model, comprehensive data on the company’s supply chain operations, including key coffee suppliers, lead times, shipping costs, and distribution centers, is gathered. Arena input analyzer is used to transform the data into the proper statistical probability distribution functions, allowing for a detailed depiction of the supply chain to identify areas for improvement and maximize efficiency. Fig 3 represents the flow chart of the company coffee SC.

The figure shows that coffee beans (CBs) are transported from the source country to the port of Aqaba in Jordan via commercial ships/ vessels in standard containers. Then CBs are unloaded at the port of Aqaba and loaded into trucks carrying 40-foot containers or two 20-foot containers to be transported to the company’s warehouses (WHs) and factory in Amman. After reclassifying and packaging, the coffee beans are transported in small trucks/ vans to the main distribution centers (DCs) or wholesalers. Then, it is transported by pickups, different means of transportation mainly private means of transportation arranged by retailers to their stores. Distribution centers are distributed in the Kingdom’s governorates and retailers are located (scattered) all over the country. Consumers buy coffee beans from the retailers’ shops.

Based on information from company data, government websites, and official trade records, the most well-known coffee exporters to the Jordanian market were identified. These resources offer a comprehensive breakdown of the nations that export coffee, facilitating an in-depth examination of the major participants in the coffee industry [5]. Table 4 identifies the main coffee suppliers for the company and their export amounts to the Jordanian market for the year 2022. This thorough data offers important insights into each supplier’s dependability and performance. The probability distributions for all the data types associated with these important suppliers, such as lead times, number and size of containers, and shipping costs, are shown in Table 5. By employing sophisticated input processing techniques to produce these distributions, the simulation model is guaranteed to faithfully replicate real-world conditions and variability. For efficient supply chain optimization and well-informed decision-making, extensive data analysis and visualization are essential.

**Table 4:** Top coffee exporters countries to Jordanian market 2022.

#	Exporter Name	Exported Quantity (Tons)
1	India	9,604
2	Ethiopia	4,487
3	Brazil	4,114
4	Nicaragua	2,491
5	Peru	949

**Table 5:** Distribution functions that characterizes each kind of data for each supplier.

Supplier	Statistical Distribution that represents each type of data			
	Lead time (Days)	No. of Containers per order	Shipping cost ($)	
			20’	40’
India	UNIF (22, 36)	POIS (2.22)	UNIF (1,527, 3,429)	UNIF (1,533, 3,911)
Ethiopia	UNIF (14, 17)	POIS (3.66)	UNIF (1,968, 2,095)	UNIF (3,385, 3,496)
Brazil	UNIF (33, 79)	POIS (4.8)	UNIF (1,197, 2,469)	UNIF (1,553, 2,607)
Nicaragua	UNIF (59, 76)	POIS (1)	UNIF (1,831, 2,157)	UNIF (1,906, 3,642)
Peru	UNIF (32, 63)	POIS (1)	UNIF (1,686, 2,122)	UNIF (1,931, 3,276)

Furthermore, several assumptions are incorporated into the simulation model to faithfully depict the logistical dynamics and guarantee consistent analysis across all suppliers and distribution scenarios. Every supplier use the same distribution function of container types; 74% of shipments use 20-foot containers, and 26% use 40-foot containers. Additionally, a consistent distribution ratio of 8.3% was expected to exist between the warehouse/ factory and its distribution centers every month. This consistency in assumptions aids in the model’s simplification while preserving its dependability for use in making decisions.

### Simulation model implementation

Arena v14 software is used for model building to enhance coffee supply chain performance, offering a reliable and user-friendly tool for developing and evaluating complex supply chain scenarios. The simulation model was run on a Dell Inspiron 5559 with a core i7 6th generation processor and 8GB RAM. This hardware configuration, compatible with Windows 11 Enterprise, provided the processing power required to run complex simulations.

The simulation model for JCoSC underwent validation and verification to ensure precision and dependability. A crucial step in the creation of simulation models is validation and verification (V&V). These procedures assurance that the model operates correctly and realistically replicates the actual system. Validation makes sure the model faithfully imitates the real-world-system, whereas verification verifies that the model is implemented correctly and error-free. V&V are crucial for guiding decision-making processes, producing accurate and realistic outcomes, and enhancing supply chain performance [56]. The verification phase ensured that all elements of the company’s coffee supply chain were included in the simulation model. The company sources from five suppliers and has a roasting plant. After roasting, the coffee is distributed to ten distribution centers within Jordan. The model also incorporates all related shipping costs, accurately representing the supply chain to achieve realistic results. During the validation phase, the model was executed based on the current state of the SC for a period of 365 days. The model’s results indicated that the company imports approximately 148 containers annually from all suppliers. This result was compared with the company’s official reports and bill of ladings, which showed an average annual import of 155 containers from 2017 to 2024 [5]. The comparison revealed an error rate of approximately 0.0452, which is less than 0.05. Consequently, the model is deemed acceptable and applicable, demonstrating its ability to accurately respond to anticipated improvements [57].

**Fig 4:**
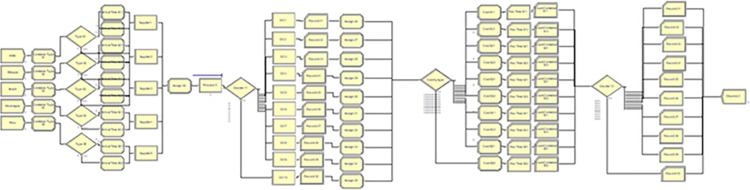
Coffee supply chain simualtion model.

Using the Arena software, a simulation model was initiated based on all of the data that had been gathered and examined. This all-inclusive model includes every essential aspect of the logistics and supply chain processes. Fig 4 illustrates a thorough visual depiction of the processes by representing different model dynamics and components.

Improving the performance of the JCoSC requires studying the available options, including the impact of configuration, studying the suppliers and means of transportation used, focusing on identifying opportunities for improvement. In this study, several scenarios will be used to measure their impact on the performance of the supply chain. The first scenario involves eliminating the worst coffee supplier based on FAHP-FTOPSIS results and transferring its quantity to the best supplier. The second scenario involves replacing the worst suppliers with new suppliers extracted from real data and ranked using the FAHP-FTOPSIS approach. The impact will be assessed by measuring lead time and cost. These scenarios will help estimate the coffee supply chain’s effectiveness and provide recommendations for improvements. Through a reduction in lead time and transportation costs, supply chain performance will be improved.

#### FMCDM integration into the simulation model.

**Fig 5:**
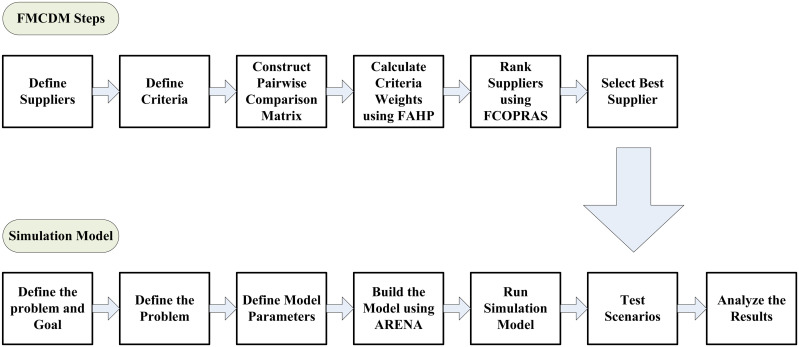
The integration of the FMCDM and the simulation model.

Fig 5 illustrates the steps of the integration of the FAHP-FTOPSIS technique in the simulation model. After arranging coffee suppliers using the integrated FAHP-FTOPSIS technique and identifying the best and worst suppliers, the ranking results are used and integrated within the simulation model to analyze and study the current JCoSC. Then, the proposed scenarios related to the results of selecting suppliers will be studied to show their effect on the performance and efficiency of the SC. The improvement achieved in the SC will be analyzed and evaluated in terms of reducing lead time and total shipping costs. Fig 5 shows the FMCDM integration with the simulation model.

Time complexity measures an algorithm’s computational efficiency and scalability, indicating the number of operations needed to run it on large datasets, crucial for optimal performance. In this study, the effect of time complexity is neglected because the datasets are not as large and complex as in other supply chains due to the limited number of sources, consistency of ordering, supply, and shipping methods, and the limited variety of coffee types compared to other products.

## Discussion of the results

This section discuss the selection and ranking of the coffee suppliers based on FAHP-FTOPSIS method, analyzing the performance of the JCoSC through the simulation model, as well as the results of integrating Fuzzy MCDM into the simulation system in different scenarios to come up with the best outcomes for improving the CoSC. The detailed analysis of the performance metrics and simulation scenarios provides suggestions for future development and improving the coffee supply chain’s effectiveness.

### Supplier selection

The Fuzzy AHP-Fuzzy TOPSIS method was used to rank the JCoSC and select the best coffee supplier from five alternatives: India, Ethiopia, Brazil, Nicaragua, and Peru. Six criteria were assessed: lead time (LT), shipping costs (SC), coffee prices (CP), coffee quality (CQ), customs tariffs (CT), and availability (AV). A sample of ten DMs is approached for a comprehensive assessment. In order to ensure a thorough and impartial assessment that would support in the decision-making process, these DM insights were essential in comparing the vendors to the established criteria. The study used rigorous validation protocols and cross-referencing from official sources to ensure data accuracy and precision. This comprehensive approach allowed for a thorough examination of shipping logistics, expenses, and Jordan’s import market, enhancing the overall accuracy and depth of the research conclusions.

#### Criteria weights.

The fifty DMs were asked to complete an evaluation matrix to assess criteria. The linguistic and crisp values were transformed into fuzzy values using the scale shown in Table 2. The average trapezoidal fuzzy matrix of the criteria weights are shown in Table 6.

**Table 6:** The average criteria weights.

Criteria	LT	SC	CP	CQ	CT	AV
Shipping	(1/3,2/5,2/3,1)	(1,1,1,1)	(2,5/2,7/2,4)	(1,1,1,1)	(4,9/2,11/2,6)	(3,7/2,9/2,5)
Coffee Price	(1/5,2/9,2/7,1/3)	(1/4,2/7,2/5,1/2)	(1,1,1,1)	(1/4,2/7,2/5,1/2)	(2,5/2,7/2,4)	(1,3/2,5/2,3)
Coffee Quality	(1/3,2/5,2/3,1)	(1,1,1,1)	(2,5/2,7/2,4)	(1,1,1,1)	(4,9/2,11/2,6)	(3,7/2,9/2,5)
Customs Tariffs	(1/7,2/13,2/11,1/5)	(1/6,2/11/2/9,1/4)	(1/4,2/7,2/5,1/2)	(1/6,2/11/2/9/1/4)	(1,1,1,1)	(1/3,2/5,2/3,1)
Availability	(1/6,2/11/2/9/1/4)	(1/5,2/9,2/7,1/3)	(1/3,2/5,2/3,1)	(1/5,2/9,2/7,1/3)	(1,3/2,5/2,3)	(1,1,1,1)

The consistency index (CI) was found to be 0.021468 ≤ 0.1 indicating the consistency of the matrix. The weights of the criteria are calculated using equations (6–8). Table 7 shows the coefficients of the criteria-weight, the summations of the 𝛾, 𝛽, 𝛾, 𝛿 and the inverse of the summation of the coefficients of the criteria weights, respectively.

**Table 7:** The coefficients of the criteria-weight.

Criteria	𝛼j	𝛽j	𝜸j	𝛿j
**LT**	1.979	2.408	3.165	3.516
**SC**	1.414	1.583	1.966	2.221
**CP**	0.541	0.639	0.858	1.000
**CQ**	1.414	1.583	1.966	2.221
**CT**	0.263	0.289	0.366	0.429
**AV**	0.361	0.419	0.558	0.458
**Sum**	5.972	6.921	8.880	9.845
**Sum Inverse** ^**−1**^	0.1674	0.1445	0.1126	0.1016

The final weights of criteria using the FAHP method are calculated using equations (9–10). Table 8 represents the final weight of criteria and the defuzzified numbers. The ranks of the evaluation criteria weight are as follows: lead time, shipping cost, coffee quality, coffee price, availability, and customs tariffs.

**Table 8:** Criterion weights.

Criteria	Trapezoidal Weights				Defuzzification Value
**LT**	0.201	0.271	0.457	0.589	0.374
**SC**	0.144	0.178	0.284	0.372	0.240
**CP**	0.055	0.072	0.124	0.167	0.1024
**CQ**	0.144	0.178	0.284	0.372	0.240
**CT**	0.027	0.033	0.053	0.072	0.0449
**AV**	0.037	0.047	0.081	0.077	0.062

### Evaluation of suppliers

DMs evaluate the JCo suppliers using the five level linguistic evaluation values. The decision makers’ linguistic evaluation of the suppliers is terraformed into TPZ fuzzy numbers, and then the average values are calculated. Table 9 shows the fuzzy evaluation vector using eq. 11–14, the crisp value and the final ranking of the suppliers using FAHP-FTOPSIS technique.

**Table 9:** The final ranking of five suppliers.

Supplier	Fuzzy Values				Defuzzified Values	Final Ranking
**India**	1.823	3.145	6.481	9.965	5.173	3
**Ethiopia**	3.321	5.061	9.618	14.006	7.781	1
**Brazil**	2.563	4.034	7.901	11.754	6.365	2
**Nicaragua**	0.993	2.024	4.591	7.627	3.642	5
**Peru**	1.375	2.551	5.494	8.781	4.374	4

Based on the supplier selection method, the best supplier is found to be Ethiopia, and the suppliers are ranked as flows: Ethiopia, Brazil, India, Peru, and Nicaragua. These results are consistent with the experts’ assessment and the real situation in the company. The results will be used within the simulation model to analyze supplier impact on the company’s coffee supply chain, aiming to optimize supplier selection and enhance overall performance. The simulation model will incorporate various scenarios to assess the company’s coffee supply chain improvements and the impact of changing suppliers.

### JCoSC Performance analysis

With the following parameters, the simulation models use the company’s coffee supply chain input data with the following simulation parameters: warm-up period of 31 days, replication length of 365 days, and the number of replications set to 30. Among the results is the number of containers for each coffee provider, the lead time, the cost of the shipment, and the overall cost of shipping. The results provide a comprehensive overview of each supplier’s performance metrics, facilitating a detailed assessment of their efficiency and cost-effectiveness. By employing the simulation output performance measures, the company may improve their supply chain and choose the best supplier depend on quantifiable facts. The primary outputs of the simulation model for each supplier are shown in Table 10. For the 20’ and 40’ containers, the shows the average lead time (days), the shipping cost ($) to ship the coffee from the supplier to Aqaba sea port in Jordan, the total number of containers required, and the total shipping costs from the origin to the destination or the consumer.

**Table 10:** Average performance measures for each supplier.

Supplier	Avg. Lead Time (Days)	Number of Containers		Total Shipping Cost ($)		
		20’	40’	20’	40’	Total Shipping Cost ($)
**India**	29	20	6	48,280	14,724	63,004
**Ethiopia**	16	60	19	121,860	63,992	185,852
**Brazil**	57	22	8	38,808	14,368	53,176
**Nicaragua**	68	4	2	6,552	6,400	12,952
**Peru**	48	5	2	8,580	3,128	11,708

The outcomes of the current situation in the company, shown in Table 10, show the performance measures for the concerned suppliers, as the company currently imports from all of these five sources in varying quantities. The results of the simulation model revealed that the best supplier in terms of lead time was Ethiopia. Shipping costs are not a sufficient indicator of the cheapness of shipping from one source to another, as these costs depend on the sea distance (since shipping is sea freight), the weight and size of the shipment, shipping conditions, sea freight party, the time of shipment, shipping line, transition (unloading and reloading of the shipment to another ship), and the geopolitical conditions of the shipping route. However, the distance between the source and the destination is considered a suitable indicator of shipping costs. The shipping quantity from each supplier depends on the availability, costs, and quality of coffee beans. Additionally, Ethiopia also had the highest performance in terms of the number of containers. The average lead time for the supply chain was 44 days for the container. The total shipping cost, of the total amount imported by the company, amounted to $326,692, distributed as $224,080 for 20-foot containers and $102,612 for 40-foot containers. Based on these results, various scenarios will be developed to further improve the supply chain performance.

### The results of the integration of FMCDM and simulation model

To improve the effectiveness of Jordan’s coffee supply chain, the model incorporates several scenarios to identify opportunities for development. The first scenario involves moving the imported quantities from the current sources to the best provider found by the FAHP-FTOPSIS method using the same procedure and data to meet market demand. This is done by eliminating the worst coffee supplier. Following this adjustment, the cost and lead time are determined. In the second scenario, the worst three coffee providers are replaced by a new suppliers selected using the FAHP-FTOPSIS methodology.

In the first scenario and based on the results of the FAHP-FTOPSIS, the worst supplier was Nicaragua, and the best supplier was Ethiopia. After removing the worst supplier from the model and compensating the demand from the best supplier, the model was run again and the output measures were taken.

**Fig 6:**
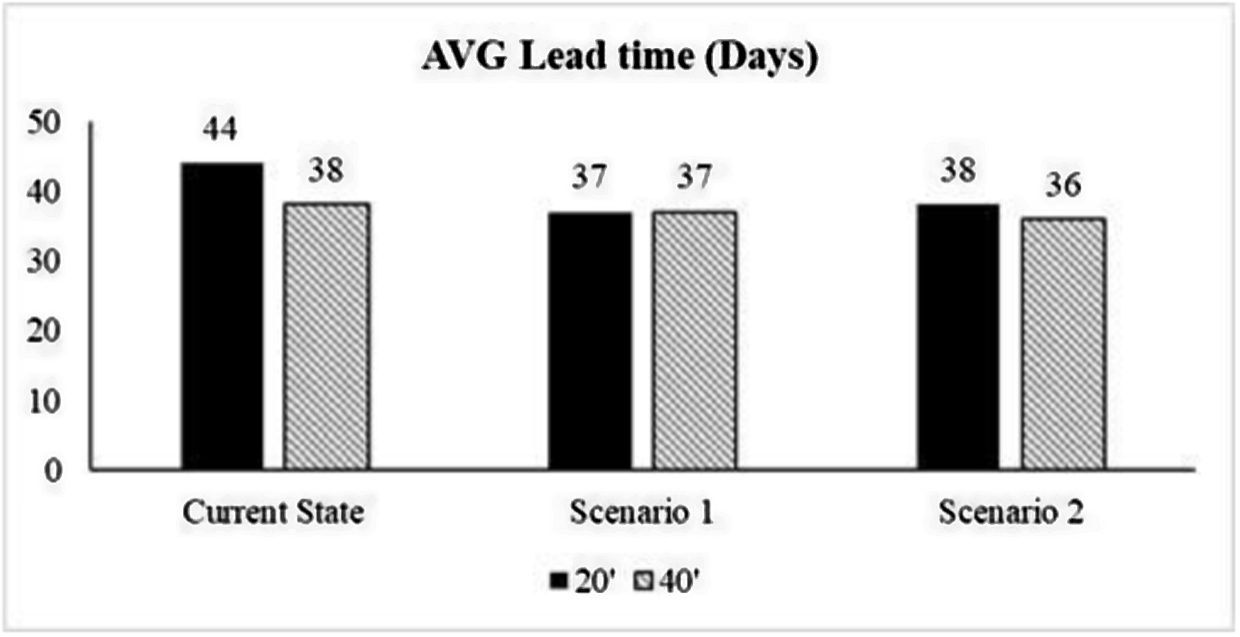
Comparison between current-state and the two scenarios in regard to average lead time.

In the second scenario, the new suppliers are selected based on expert opinion and ranked based on the results of the FAHP-FTOPSIS method. The proposed suppliers were Colombia, Kenya, and Vietnam. Kenya was found as the best supplier followed by Colombia then Nicaragua. The new suppliers are then integrated into the simulation model, replacing the worst suppliers (India, Peru, and Nicaragua), and the model was executed again based on these new parameters. The following Fig 6 illustrate the key results of the first and second scenarios and compare them with the current state of the supply chain.

**Fig 7:**
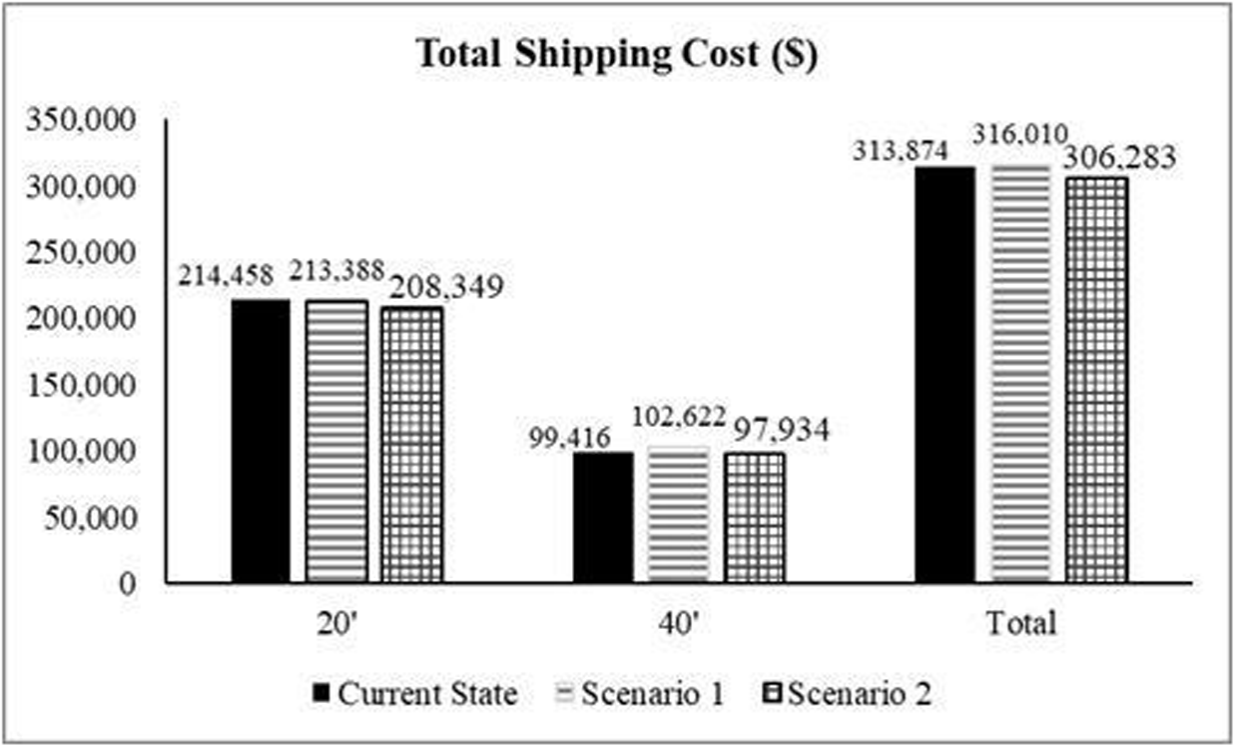
Comparison between current-state and the two scenarios in regard to total shipping cost.

**Fig 8:**
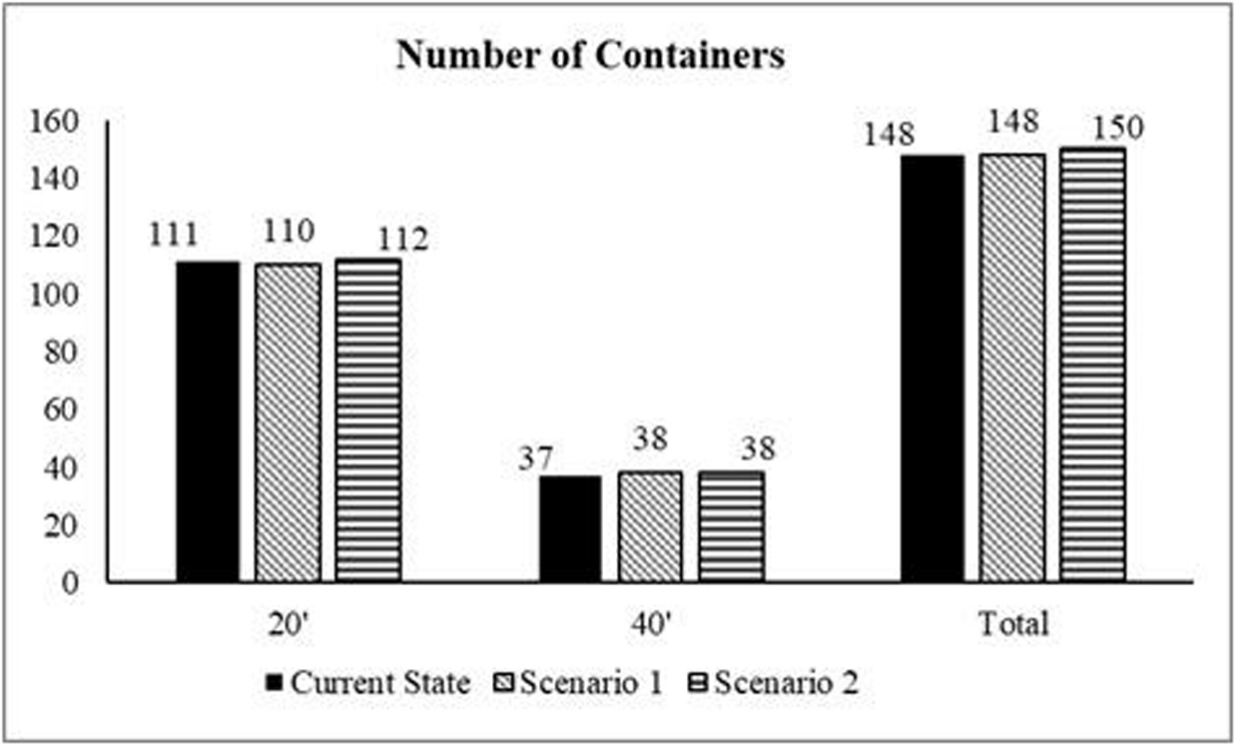
Comparison between current-state and the two scenarios in regard to number of containers.

Depending on the analysis of the scenarios’ outcomes and their comparison with the current state of the JCoSC, it was found that both scenarios reduce the average lead time. However, while the first scenario increases the total shipping costs, the second scenario reduces the total shipping costs. Additionally, the second scenario resulted in an increase in the total number of containers imported by the company to 150, despite the lower total shipping costs and reduced average lead time. Therefore, the final recommendation to improve the performance of the company CoSC is to cease importing from Nicaragua and start importing from Kenya as a new supplier. This change ensures lower shipping costs, shorter lead times, and an overall improve in the efficiency of the coffee SC.

## Conclusions

One of the most consumed drinks in the world is coffee. Today’s adults still consume a lot of coffee, the majority drinking one or more cups each day. The agri-food SC is important because it meets people’s needs on a daily basis. The coffee supply chain is of special consideration because it has become a daily habit and a beverage that most people drink on a daily basis, among most peoples of the world. Despite the multiple sources of coffee beans, it has become a critical product due to the increasing demand for this product. Coffee is considered one of the important drinks in Jordan and has historical and tribal considerations and social symbolism, as it is used in all public and private occasions. In addition, it is one of the drinks served in hospitality and is drunk several times a day. Coffee has many types and is used in many food products.

This study considers selecting the best coffee supplier and improving JCoSC performance in Jordan through fuzzy MCDM and DES models provides insights into improving Jordan’s coffee SC’s efficiency and effectiveness. The research’s findings offer a widely overview of CoSC’s current situation and suggest opportunities for development. The tools utilized make it easier to rank the coffee suppliers and optimize the CoSC while maintaining coffee quality. Through a methodical assessment of supplier performance, the best and least favorable suppliers were identified. The simulation model provided further details on each supplier’s performance indicators, including lead time, shipping costs, total shipping expenses, and the number and type of imported containers. Ethiopia demonstrated leadership in several indicators, indicating that the business might decide to collaborate with it as a preferred supplier. Scenario analysis using simulation models also brought to light the possible advantages of switching from subpar to superior suppliers.

The research’s conclusions highlight the importance of using advanced methods like the integration of FAHP-FTOPSIS results within the simulation model to optimize SC operations. The findings of the research provide the company and other coffee industry and stakeholders with practical advice on how to improve CoSC effectiveness, cut costs, and improve overall efficiency. Jordan’s coffee supply chain requires developments to improve efficiency. Reducing lead time and shipping costs by switching to African suppliers, investing in technology like real-time tracking systems and management software, and establishing a culture of continuous improvement are recommended. These measures will ensure stability and quality of imported coffee, improve decision-making, and adapt to market conditions.

The findings of the study can be used by those involved in coffee supply chains to select and arrange suppliers and improve performance. The study has a number of limitations, including the use of a single company, limited number of suppliers, the adoption of a specific number of criteria, and consideration of few metrics like shipping costs and lead time. Future works may include other criteria, more suppliers, and consideration of the costs of the entire supply chain, in addition to agricultural and social considerations, changing environments, and SC disruptions. Future studies may include warehouses, distribution centers, ordering techniques, distribution methods, and inventory related to the coffee supply chain.

Further investigation may include taking advantage of new growth opportunities and reduce market volatility risks by diversifying suppliers from different areas; Considering long-term sustainability planning involves developing strategies that prioritize social responsibility, climate change resilience, and ethical sourcing to ensure sustainable practices; And studying the implementation and monitoring of innovative CoSC solutions to study their effects over time. The study could be extended to other companies and countries to ensure its generalizability.

Even though the research has shed light on how to enhance Jordan’s coffee supply chain, there are still a number of areas that might require more investigation and development. Future research could explore additional criteria for supplier selection and supply chain optimization, considering elements like geopolitical stability, reliable sourcing, and environmental sustainability. By adding more intricate elements to the existing models—like demand unpredictability, production limitations, and SC risks it is possible to get a better understanding of supply chain dynamics and pinpoint possible areas for development. To promote innovation and ongoing development in the coffee supply chain, collaborative research projects combining academic institutions, business partners, and governmental organizations can help exchange best practices and knowledge.
